# The impact of COVID-19 on O&G trainees; where are we now?

**DOI:** 10.52054/FVVO.14.1.007

**Published:** 2022-04-03

**Authors:** I Duggan, R Hablase, L Beard, F Odejinmi, R Mallick

**Affiliations:** Conquest Hospital, East Sussex Healthcare NHS Trust, The Ridge, St Leonards-on-Sea, East Sussex, TN37 7RD; Princess Royal Hospital, University Hospitals Sussex NHS Foundation Trust, Lewes Road, Haywards Heath, RH16 4EX, UK; Whipps Cross Hospital, Barts Health NHS Trust, Whipps Cross Road, Leytonstone, London, E11 1NR, UK

**Keywords:** COVID-19, coronavirus, training

## Abstract

**Background and Objectives:**

Obstetrics and Gynaecology (O&G) training continues to face challenges caused by the COVID-19 pandemic, particularly in gynaecological surgical training. This follow-up survey captures the ongoing effect on O&G trainees and highlights the future recovery plan considering the historical training gaps in benign gynaecology.

**Materials and Methods:**

an anonymised survey was emailed to all O&G trainees in Kent, Surrey and Sussex (KSS). Responses were collected over 6 weeks.

**Main Outcome Measures and Results:**

53% of trainees responded. In total, 78% of trainees agreed that the pandemic had an ongoing negative effect on their physical and mental wellbeing respectively. Trainees felt the prior negative impact on obstetric training is improving, whilst 88% still experience a negative impact on their gynaecology surgical training despite the resumption of elective services in the National Health Service (NHS). 80% continue to feel the negative impact on their educational activities and 88% felt their overall training continues to be negatively impacted. 70% were positive that they would recover from this. Responses were representative of each training year. Interestingly, 95% of trainees had accepted the COVID vaccine.

**Conclusion:**

despite “restoration” of normal services, the negative impact on trainees particularly benign gynaecology surgical training continues. Addressing pre-pandemic training gaps whilst tackling the surgical back- log and the needs of service provision will continue for years to follow.

**What is new?:**

Future training needs to incorporate creative ways of acquiring surgical skills. It is imperative to imbed simulation training into O&G training programmes. Pastoral support is key to ensure trainees’ mental and physical well-being are prioritised and the already high burn-out rates do not worsen.

## Background

The COVID-19 pandemic has had a devastating impact on healthcare systems worldwide and its negative impact continues to be felt despite the attempted restoration of “normal” services (Mallick et al., 2020; Odejinmi et al., 2020,). Patient waiting times for benign gynaecological surgery continue to grow and the overall impact on gynaecological services will undoubtedly be felt for many years. Trainees have also been disproportionately negatively impacted and similarly these negative effects may be felt for many years to come. Many trainees were redeployed to support front line services at the height of the pandemic despite not feeling clinically competent to do so and many reported a significant effect on their mental and physical wellbeing (Mallick et al., 2021). Furthermore, training opportunities available in the outpatient and surgical settings were drastically reduced due to the cessation of face- to-face teaching, clinical consultations, and non- emergency surgery. A recent survey highlighted the immensely disruptive impact of COVID-19 on O&G trainees with almost all trainees stating a significant negative impact on their benign gynaecology surgical training (Mallick et al., 2021). These concerns were all highlighted at the peak of the pandemic and despite the restoration of normal services, training continues to be impacted. New challenges are also now being faced including the outsourcing of elective surgeries to the independent sector, the slow recovery process and overall lack of surgical exposure. This coupled with the limited face -to- face educational opportunities, the ongoing pressures to meet yearly surgical competencies and the historical challenges facing gynaecology surgical training continues to significantly impact the training of the next generation of gynaecologists.

As the COVID-19 pandemic continues to evolve, the aim of this follow-up survey was to assess the ongoing impact of the COVID-19 pandemic on the experiences of O&G trainees following the pandemic peaks. The experiences explored within this survey include training opportunities, surgical practices and the effects of these unprecedented times on the mental and physical wellbeing of the trainees as well as the perceived long-term impact of the COVID-19 pandemic on training and career pathways.

## Methods

All O&G trainees within the Kent, Surrey and Sussex (KSS) region were sent an email invite to undertake an anonymous 36-question survey. Each set of 2 to 4 questions focused on a training aspect. Topics explored included mental and physical wellbeing, redeployment, obstetric training, gynaecology surgical training, annual training outcomes, teaching and educational opportunities. The answers ranged from strongly agree to strongly disagree (a total of five options). The last 3 questions invited comments and ideas to support training moving forward. Informed consent was gained via the initial email invite and the data was collected over a 6-week period. The data collected was compared with data previously collected and published (Mallick et al., 2021). A combination of positive and negative questions in a non-consequence manner were utilised to minimise potential bias. Data was analysed using SPSS version 27. Thematic analysis of the data including trainee comments was also undertaken.

## Results

Of the 127 O&G trainees currently in programme within the KSS region, 67 responded giving a 53% response rate. The demographics of the respondents are summarised in Table I.

**Table I:** Respondent demographics.

Demographic	% (n)
Training grade	
ST1	11.94 (8)
ST2	13.43 (9)
ST3	13.43 (9)
ST4	23.88 (16)
ST5	16.42 (11)
ST6	10.45 (7)
ST7	7.46 (5)
Subspec trainee	2.99 (2)
Age	
18 to 24	0
25 to 34	56.06 (37)
35 to 44	40.91 (27)
45 to 54	3.03 (2)
55 to 64	0
Over 65	0
Gender	
Male	5.97 (4)
Female	92.54 (62)
Non-Binary	0
Prefer not to say	1.49 (1)
Ethnicity	
White	53.03 (35)
Mixed	9.09 (6)
Asian	22.74 (15)
Black	6.07 (4)
Any other ethnic group	1.52 (1)
Prefer not to disclose	7.58 (5)

18.97% (11/58) and 60.34% (35/58) of trainees agreed or strongly agreed that the COVID-19 pandemic continued to have a negative impact on their physical and mental wellbeing respectively. 95.31% (61/64) of trainees had been offered and accepted the COVID-19 vaccine. 1.56% (1/64) had not been offered the vaccine and 3.13% (2/64) had declined the vaccine.

### Work and training

After the first wave, 3.13% (2/63) were redeployed to cover areas outside O&G and of those 50% felt clinically competent to do so. 11.21% (13/58) agreed or strongly agreed that the COVID-19 pandemic continues to negatively affect their obstetric training experience compared to 88.14% (52/59) who felt their benign gynaecology surgical training experience continues to be negatively affected.

**Figure 1:**
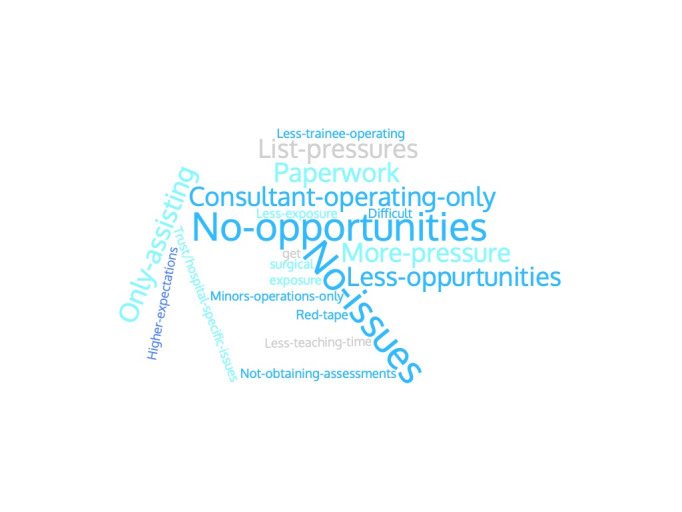
Trainee responses to: ‘‘Have you experienced any issues with regards to operating in the independent sector?

Only 13.55% (8/59) agreed or strongly agreed that operating in the independent sector (IS) was a feasible alternative to gaining surgical competencies. Perceived barriers to operating in the IS are summarised in Figure 1. 20.34% (12/59) agreed simulation training had been a feasible alternative to gaining surgical competencies.

66.1% (39/59) agreed or strongly agreed that their emergency gynaecology surgical training continues to be negatively impacted by COVID-19. 18.64% (11/59) had met their surgical competencies over the past year and 93.22% (55/59) felt their surgical exposure was lower or significantly lower compared to pre-pandemic.

15.25% (9/59) and 74.57% (44/59) of trainees agreed or strongly agreed that their antenatal clinic and gynaecology clinic experiences respectively, continue to be negatively affected. 35.59% (21/59) and 47.46% (28/59) agreed or strongly agreed that their obstetric scanning and gynaecology scanning experience continues to be negatively affected.

### Long term impact and career pathway

Almost 80% of O&G KSS trainees felt their educational activities continue to be negatively impacted by COVID-19. 88.14% (52/59) felt that COVID-19 had negatively impacted their overall training over the past 12 months. 32.2% (19/59) reported a negative impact on their Annual Review of Competence Progression (ARCP) outcome due to COVID-19 with 5.17% (3/58) having to extend their training time.

29.31% (17/58) agreed or strongly agreed that COVID-19 had negatively impacted on their ATSM choices with 34.48% (20/58) agreeing that the COVID-19 had forced a necessary, but unwanted change in career plans. 70.69% (41/58) were positive they would recover from the negative impacts of COVID-19.

Table II summarises the trainee responses to the wellbeing and training questions comparing both the first and second survey.

**Table II:** Trainee responses regarding wellbeing, training and future.

	First Survey			Follow up survey		
	Strongly agree/agree (%)	Neither agree nor disagree (%)	Strongly disagree/disagree (%)	Strongly agree/agree (%)	Neither agree nor disagree (%)	Strongly disagree/disagree (%)
Wellbeing						
The COVID-19 pandemic has affected your physical wellbeing.	36	20	41	19	19	62
The COVID-19 pandemic has affected your mental wellbeing	77	5	18	60	12	28
Training						
COVID-19 has negatively affected your obstetric clinical training experience.	43	16	41	22	21	57
COVID-19 has negatively affected your obstetric antenatal training experience.	46	15	39	15	27	58
COVID-19 has negatively affected your obstetric ultrasound experience.	61	26	13	35	29	35
COVID-19 has negatively affected your gynaecology ultrasound experience	-	-	-	47	32	20
COVID-19 has negatively affected your gynaecology outpatient training experience.	93	3	4	74	8	17
COVID-19 has negatively affected your emergency gynaecology surgical training experience.	84	9	7	66	17	17
COVID-19 has negatively affected your benign gynaecology surgical training experience.	99		0	88	7	5
I have met my gynaecology surgical training competencies this year	-	-	-	19	13	68
Long term impact and career pathway						
My educational opportunities have been negatively affected by COVID-19	93	5	2	80	9	12
I am concerned about the impact of COVID-19 on my overall training.	79	15	6	88	7	5
COVID-19 will likely have/has had a negative impact on my training progression	56	29	15	88	7	5
COVID-19 had a negative impact on my last ARCP outcome	-	-	-	32		68
COVID-19 impacted on my ATSM choices	-	-	-	29	46	24
COVID-19 resulted in an unwanted, but necessary change in my career/training plans	-	-	-	34	34	31

**Figure 2:**
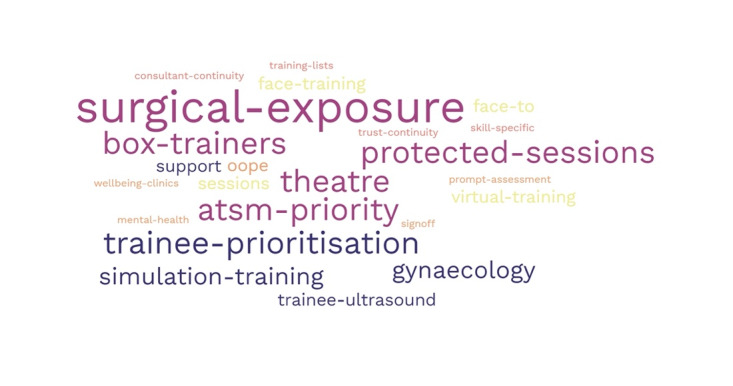
Trainee responses to ‘how they could best be supported to minimise the impact of the pandemic moving forward.

Figure 2 summarises the main themes when trainees were asked to comment on how they could best be supported to minimise the impact of the pandemic moving forward.

## Discussion

As we work through the second year of the COVID-19 pandemic, concerns over the mental health wellbeing of healthcare professionals remains significant. Long-term studies highlighted the persistence of depression and anxiety disorders in frontline HCP’s with almost 10% reaching post- traumatic stress (PTS) threshold scores. (Chan et al., 2020). The survey shows similar trends among KSS trainees with 60% agreeing or strongly agreeing to the on-going negative impact on their mental wellbeing.

Stressors such as recurrent and prolonged self- isolation, taking time off as a carer, having family members affected, delayed milestone professional exams, a drastic reduction in operative workload and the uncertainty about training progression are some of many reasons leading to prolonged mental health sequalae. Failure to ensure HCP’s well-being can lead to burnout, poor patient care (Robson and Cukierman, 2019), more medical errors and higher absenteeism (Shanafelt et al., 2010). Furthermore, O&G historically is a profession with a high burnout rate, and this is likely to worsen due to COVID- 19 pandemic (Bourne et al., 2019; RCOG 2020a). Peer support, mentorship and regular supervisor meetings are recommended to mitigate against this. Formal pastoral care support may also be beneficial to educational supervisors enabling a confidential and structured way to discuss workplace and personal stressors (RCOG 2020a). Reassuringly the negative physical wellbeing impact of COVID-19 has reduced from 39% to 19%.

The pandemic had fundamentally changed the daily practice of medicine in three ways: telemedicine was rapidly adopted; hospitals increased their threshold for urgent admissions and planned elective procedures were halted (Ferriss et al., 2021). Some of these practices, in particular virtual consultations, are likely to remain the “norm” for many years to come and may inadvertently have a negative impact on gynaecological training (NHS England 2019– NHS long term plan). This is certainly reflected in this survey; 74% of trainees felt that their gynaecological outpatient training experience has been negatively impacted. Full restoration of face-to-face gynaecological consultations with trainees prioritised to attend are essential to ensure gynaecological competencies are met.

Obstetric training appears to have made a more rapid recovery or perhaps due to the urgent and dynamic nature of obstetrics combined with ‘business as usual’ for most obstetric activity, trainees feel their training is overall less impacted. The number of trainees who felt their obstetric antenatal clinic experience had been negatively impacted dropped from 46 to 15%. The overall perceived negative impact on obstetric training also reduced from 43 to 22%.

Despite the resumption of normal elective gynaecological activity, it remains below pre- COVID levels. In July 2021 there were still 17% fewer admitted treatments and 15% non-admitted treatments (House of commons Library, NHS Key statistics: England, October 2021). It is estimated that cancelled elective surgical procedures during the 12-week peak of COVID-19 in Spring 2020, sets at 81.6% for benign gynaecology (Ball et al., 2021). Moreover, the pool of patients awaiting surgery is growing with the accelerated resumption of hospital out-patient activities to clear the back log of referrals. The pressure to recover from the pandemic disruption jeopardise training opportunities in many ways. 88 % of the trainees feel that their benign gynaecology surgical training is being negatively impacted. Despite the slight improvement from 99% in the previous survey, training gap is still significant. In this survey only 19% of trainees felt they had met their surgical competencies in the past year and 93% felt their gynaecological surgical exposure was lower or significantly lower compared to pre-pandemic.

This is not entirely unexpected, considering that gynaecological surgical training had always fallen behind the opportunities in obstetrics pre-pandemic. The 2019 Royal College of Obstetricians and Gynaecologists (RCOG) training evaluation survey highlighted that only 59.4% of trainees fulfilled their training requirements in gynaecology. The perceptions of trainees on gynaecological training in 2018 and 2017 reflected the ongoing challenges pre-dating the pandemic and are summarised in Table III below.

**Table III:** RCOG 2017-2018 training evaluation survey.

	2017	2018
Adequate opportunity to fulfil training requirements in Gynaecology	58.5%	56%
Access to laparoscopic box trainers/virtual reality simulators	53.4%	54%
Formal programme of simulation training in Gynaecology	16.7%	19%

The statements from Health Education England (HEE, 2021) and the recently published RCOG training plan (RCOG, 2021) echo the problems highlighted and gave suggestions on how to progress gynaecology training. Several potential strategies were suggested such as involving local NHS trusts and acknowledging training difficulties in their risk registers, structured national simulation programmes and the development of “trainee centred training” based on their career level and future plans.

Trainee centred training should encompass regular supervisor meetings, simulation training and pastoral support. Ideally all trainees within the next 12 months should have a 1:1 meeting with their training programme directors (TPDs) to assess training deficits, create a structured and achievable training plan and strategize future placements to ensure sufficient workload depending on their training needs. (RCOG,2021; HEE,2021)

The gaps in gynaecological surgical training have clearly been worsened by the COVID-19 pandemic and will not only impact on the rate and time of skill acquisition amongst trainees, but it may also potentiate “surgical-skill decay”. That is more likely to be the case for higher trainees and junior consultants as many will experience loss of acquired skills after a period of non-use. Hoopes et al. (2020) highlighted the benefits of home surgical training resources for O&G trainees during the pandemic. Examples include Laparoscopic box trainers, virtual reality trainers, homemade simulation models, video games, online surgical simulation, webinars, smart phone applications and surgical videos (Green et al., 2019). Furthermore, the British Society of Gynaecological Endoscopy (BSGE) has recently initiated a structured centralised programme delivered through a series of hands-on workshops combined with online webinars across the country.

The impact of the pandemic on higher trainees is a particular concern with 29% stating that the COVID-19 pandemic had negatively impacted their advanced training skill module (ATSM) choices. Prioritising training opportunities for senior trainees and a clear recovery plan with, if deemed required and beneficial, a planned non penalised extension to training time for this group of trainees is paramount. Supporting the trainees who had to make unwanted, yet necessary career changes should not be overlooked as our survey highlighted that a third of the respondents had to make such detrimental decisions.

These potential solutions need to continue beyond the acute COVID-19 recovery period and be truly embedded into the O&G training programme. Out of programme experiences (OOPE), clinical fellowships and post certification of completion of training (CCT) clinical fellowships should be encouraged and historical barriers to access these removed. As the trainees affected by the pandemic become consultants it will be necessary to continue to support them, creating job plans which allow time for additional gynaecology training. This has been recognised by the RCOG and funds have been pledged to aid recovery from the department of health and HEE.

The widespread acceptance of the COVID-19 vaccine is a major step in the pandemic elimination strategy with 95% of respondents in our survey having had the vaccine. However, globally we still face many challenges including virus mutations and temporary outbreaks through border control failures (Baker et al., 2020). Careful interpretation of the situation along with developing surgical training methodology is essential to continue to deliver effective training despite the evolving challenges.

As with the first survey of KSS trainees we acknowledge that this is a small sample. The reduction in response rate when compared to the initial survey (69% to 53%), may reflect that although the perceived impact on their physical and mental wellbeing is still high, it is less than that experienced in the first wave. Furthermore, there may be an element of COVID and survey fatigue. The potential response bias of those most negatively impacted being more likely to respond also remains and highlights the need to assess the overall training impact on a wider European and/or global scale.

Whilst we acknowledge that the first and second surveys predominantly focussed on the negative impacts of the pandemic on O&G training, trainees highlighted some positive opportunities born entirely from the unique situation the pandemic has posed. In particular 84% of respondents felt that webinars and virtual training opportunities were a positive outcome. In addition, 70% felt optimistic that they would recover from the negative impacts on their training. Undoubtedly the pandemic will have enhanced our skills with prioritisation, team working, management of crisis situations and adaptability.

## Conclusion

This follow-up survey continues to highlight the ongoing challenges of the COVID- 19 pandemic on O&G training. Whilst obstetrics training seems to be recovering well the situation is vastly different for gynaecological training particularly the surgical aspect. Trainees continue to be concerned as to how they will obtain the appropriate gynaecological surgical skills, how COVID-19 will impact their ATSM choices and the ability to complete surgical ATSMs and the effect on overall career planning. As we continue to emerge from the COVID-19 pandemic new challenges will be faced balancing tackling the surgical backlog whilst safeguarding surgical training and optimising surgical opportunities. Individualised training needs must be assessed on a regular basis and tailored training plans coordinated by TPDs essential. Building a robust gynaecological training programme supported by the RCOG framework with simulation training is also key. Understandably the mental and physical wellbeing of trainees and continuous pastoral support is also paramount.

Surgical training is clearly a hands-on experience and no doubt that repetitive practice of the surgical techniques is important for the trainee to become more efficient and confident. However, lessons learnt from the COVID-19 pandemic resulted in creative adaptation of new techniques to contemporise the medical paradigms. Virtual reality and simulation training amongst many other home surgical training resources are evolving and soon will be a fundamental part of the future surgical training. In part, this will allow the acquisition or maintenance of basic surgical skills and is anticipated to expedite the trainee learning curve. The pandemic challenges have made us more creative and hopefully in the long term more efficient in the way we teach and learn, and this can only benefit us in the future and safeguard the training of the next generation of gynaecologists.
